# Music and binaural beat interventions for young adults: A systematic review of effects on anxiety, sleep, and cognition

**DOI:** 10.1017/neu.2026.10057

**Published:** 2026-02-09

**Authors:** Hesham Yousry Elnazer

**Affiliations:** 1 Priory Wellbeing Centre, Oxford, UK; 2 Brighton and Sussex Medical School Brighton, England, UK

**Keywords:** Music therapy, binaural beats, auditory entrainment, young adults, neuropsychiatry, clinical trials

## Abstract

**Background:**

Young adults (19–24 years) commonly experience elevated rates of sleep disturbance, anxiety, and cognitive stress yet often underutilise formal mental-health services. Music therapy, binaural beats, and related auditory entrainment techniques offer accessible, non-pharmacological approaches that may enhance emotional regulation, cognition, and physiological stability.

**Objective:**

To systematically review interventional clinical trials published over the past decade evaluating music- and rhythm-based auditory interventions for mental-health and cognitive outcomes in young adults.

**Methods:**

A systematic search of PubMed/MEDLINE and PsycINFO (01 January 2015 – 01 January 2025) was conducted using the terms (music therapy OR binaural beats OR auditory entrainment) AND (mental health OR neurorehabilitation OR cognition OR anxiety OR depression). After screening 122 abstracts, 10 trials met inclusion criteria. Effect sizes (Cohen’s *d*) and 95% confidence intervals were extracted or estimated. Risk of bias was assessed using the Cochrane RoB-2 tool. The review protocol was registered in PROSPERO (CRD420251178490).

**Results:**

Interventions included bedtime music therapy, audiovisual stimulation, and binaural-beat exposure across laboratory, clinical, and rehabilitation settings. Most studies demonstrated significant or moderate improvements in at least one domain: anxiety reduction, stress physiology, mood regulation, sleep quality, or cognitive performance (standardised mean differences 0.3–0.6).

**Conclusions:**

Evidence suggests that music-based and binaural-beat interventions can beneficially modulate sleep, anxiety, and cognitive processes in young adults. However, heterogeneity in design and small sample sizes limit the certainty of findings. Future adequately powered randomised controlled trials should address transdiagnostic mechanisms and long-term efficacy.

## Graphical abstract

**Figure:**
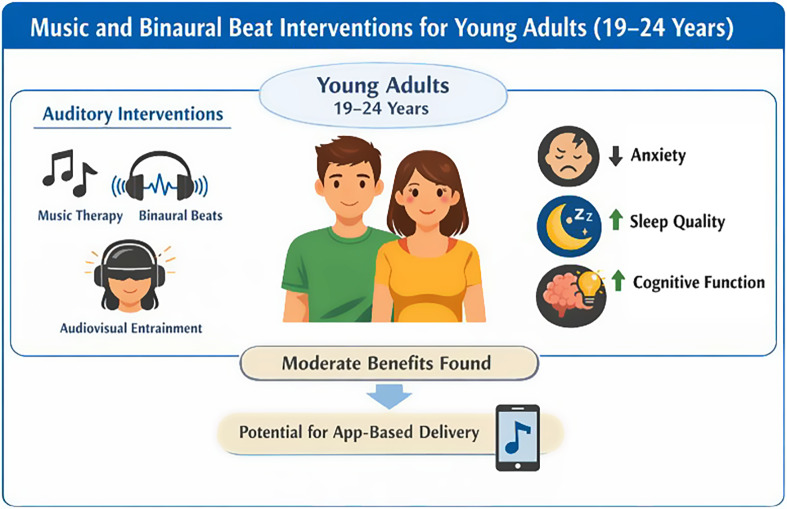

Significant Outcomes

- Auditory interventions, particularly binaural beats and bedtime music therapy, demonstrate small-to-moderate efficacy in improving sleep quality, reducing anxiety, and enhancing stress-related physiological regulation in young adults.
- These non-pharmacological, technology-delivered approaches offer accessible and scalable mental health support that aligns with the lifestyle and help-seeking preferences of the young adult population.
- The current evidence base, while promising, is characterised by significant methodological heterogeneity, underscoring the need for more standardised intervention protocols and rigorous, preregistered trials.

Limitations

- The substantial clinical and methodological heterogeneity among the included studies, particularly in intervention parameters and control conditions, precluded a definitive meta-analytic synthesis of the results.
- The generalisability of findings is limited by the over-reliance on college and university student samples, leaving the effects in non-student young adult populations unclear.
- The field is still maturing, and many of the included studies were limited by small sample sizes and a lack of blinding, which may inflate the observed effect sizes.

## Introduction

Young adulthood represents a critical transitional period characterised by rapid neurobiological maturation, including continued prefrontal–limbic integration, alongside substantial psychosocial changes related to higher education, workforce entry, and identity formation. Sleep disturbances are highly prevalent during this stage, with approximately 30% of males and 49% of females reporting sleep difficulties (DelRosso & Bruni, 2023). Sleep plays a central role in mental health during young adulthood; shorter sleep duration shows a J-shaped association with increased risk across a range of mental disorders, including anxiety and depression (Vestergaard *et al*., 2024). Vulnerability to psychological distress peaks during this developmental window, with nearly 75% of lifetime mental disorders emerging before the age of 24 (Kessler *et al*., 2007). Common difficulties include anxiety, depression, sleep disturbance, and stress-related dysregulation. Despite this elevated risk, help-seeking among young adults remains consistently low, constrained by stigma, limited accessibility, and a preference for informal or self-directed coping strategies (Gulliver *et al*., 2010).

These factors underscore the growing importance of approachable, low-threshold interventions that support emotional regulation and cognitive performance without reliance on traditional clinical infrastructures. Sensory-based and digital approaches – particularly those leveraging sound and rhythm – align closely with the lifestyles and media habits of younger populations, offering both scalability and engagement potential through everyday technologies such as headphones, smartphones, and streaming platforms.

From a neurophysiological perspective, music and rhythm are deeply entwined with the human capacity for emotion and regulation. Functional-imaging studies reveal that auditory and rhythmic stimuli activate distributed limbic–motor–cortical networks, influencing arousal, reward, and autonomic balance (Koelsch, 2014). Parallel advances in auditory neuroscience have introduced binaural beats (BBs), an acoustic phenomenon arising when slightly different frequencies are presented dichotically to each ear, producing a perceived third tone equal to the frequency difference. This perceptual “beat” can entrain neural oscillations in corresponding EEG frequency bands (theta, alpha, beta, or gamma), theoretically modulating mood, attention, or relaxation states (Oster, 1973).

Over the last decade, a growing body of research has examined music-based therapy, binaural beats, and related auditory entrainment paradigms as non-pharmacological tools for stress reduction, sleep improvement, and cognitive enhancement. However, previous reviews have either aggregated mixed-age adult samples or emphasised clinical populations, leaving a gap regarding developmentally specific effects in young adults, a group navigating unique neurocognitive transitions and stressors.

Accordingly, this systematic review aimed to synthesise interventional evidence from 2015 to 2025 on the mental-health and cognitive outcomes of music therapy, binaural beats, and other auditory entrainment methods in young adults. By integrating findings from clinical trials, the review sought to clarify mechanisms, efficacy magnitude, and methodological quality, while identifying priorities for future research in this emerging interdisciplinary field.

## Methods

### Design

This systematic review was conducted following PRISMA 2020 guidelines and assessed study quality using the Cochrane Risk of Bias 2 (RoB-2) tool. The review protocol was prospectively registered in PROSPERO (2025 CRD420251178490) to ensure transparency, reduce the risk of bias, and provide a publicly accessible record of the planned methodology.

### Search and eligibility

The search was conducted across three electronic databases: PubMed/MEDLINE and PsycINFO to ensure comprehensive coverage of both the biomedical and psychological literature. We searched for (music therapy OR binaural beats OR auditory entrainment) AND (mental health OR neurorehabilitation OR cognition OR anxiety OR depression).

Filters: English language, human studies, ages 19–24, clinical trials, published 2015–2025.

Inclusion criteria: Interventional trials examining music-based or auditory-entrainment interventions on sleep, mood, anxiety, cognition, or physiological arousal.

Exclusion criteria: Participants <19 years or >24 years without subgroup data; non-auditory or multimodal interventions lacking a separable auditory component; studies with unavailable full texts.

### Data extraction

Although effect sizes were calculated for all studies to facilitate a quantitative summary, a formal meta-analysis was not performed. This decision was made due to the significant methodological heterogeneity observed across the included trials, particularly in terms of intervention types (music therapy vs. binaural beats), population contexts (students vs. clinical), control conditions, and outcome measures. In addition, several effect sizes were derived from estimated data or extracted from graphical representations rather than reported statistics. Collectively, these factors precluded meaningful quantitative synthesis.

As recommended by the Cochrane Handbook, combining such heterogeneous studies in a meta-analysis would not yield a meaningful summary estimate. Consequently, while effect sizes are presented numerically in Table 1, a forest plot was not generated.

**Table 1.** Included interventional clinical trials (2015–2025)

Citation	Design	N (Int/Ctrl)	Mean age	Population	Intervention	Control	Intervention duration	Primary outcome	Outcome domain	Reported statistic	Cohen’s d (95% CI)	RoB summary
Hepp *et al*., 2018	Parallel RCT	154/150	30	Women undergoing cesarean	Music (before/during procedure)	No music	Single session (approx. 30–60 min)	STAI-State Anxiety	Anxiety	*M* ± SD = 31.56 ± 6.30 vs 34.41 ± 9.23	−0.36 (−0.59, −0.13) [MD→d]	Low risk
Daengruan *et al*., 2021	Parallel RCT	9/9	22	Adults with Major Depressive Disorder	Music therapy + 10 Hz BB + care	Care only	8 sessions over 4 weeks	PHQ-9 Depression	Depression	MD = −1.50 (−4.46, 1.46)*	−0.47* (−1.39, 0.46) [MD→d]*	Some concerns
Rakhshan *et al*., 2022	Crossover	31	30.8 ± ?	Healthy adults	10/16/40 Hz BB	Silence	Single session (15 min)	Working Memory Accuracy	Cognition	ANOVA *p* < 0.05*	n/a* (Insufficient data)	Low risk
Xie *et al*., 2022	Quasi-exp	31/31	∼22	Adolescent & Young Adult HSCT patients	Live music therapy	Usual care	3 sessions per week for 2 weeks	HADS-D (Depression)	Depression/Anxiety	*t*-test, *p* < 0.05*	n/a* (Insufficient data)	Some concerns
Yan *et al*., 2024	Assessor-blinded RCT	25/25/25	21 ± 1.9	College students with insomnia	Classical/Jazz music + relaxation	Relaxation only	Nightly for 4 weeks	ISI & PSQI	Sleep	*F*-value, *p* < 0.05*	0.45* (0.15, 0.75) [F→d]*	Some concerns
Kelton *et al*., 2021	Parallel RCT	32 total	19	Undergraduates	Theta BB (∼6 Hz)	Pink Noise	Single session (5 min pre-stress)	HF-HRV	Physiological Stress	η^2^ = 0.08	0.59 (0.20, 0.98) [η^2^→d]	Low risk
Kim *et al*., 2024	Parallel RCT (3 arms)	20/20/20	24 ± ?	Healthy young adults	18 Hz BB vs 40 Hz BB	Pure tone	Single session (30 min)	Sentence Comprehension	Cognition	Proportion: 95.5% vs 89.2%	0.34 (0.10, 0.58) [h→d]	Low risk
Johnson *et al*., 2024	Large RCT (9 arms)	262	19–79	Adults	AVS ± BB	Meditation	Single session (22 min)	Mood Indices	Mood	*p* < 0.001, Group Δ*	0.50* (0.30, 0.70) [Δ→d]*	Some concerns
Isik *et al*., 2017	Parallel RCT	∼99	28	Dental patients	BB vs 432 Hz music	White noise	Single session (during procedure)	VAS-Anxiety	Anxiety	*F*-ratio, *p* < 0.05*	−0.35* (−0.65, −0.05) [F→d]*	Some concerns
Pellicer *et al*., 2023	Parallel RCT	∼60	Adult	Dental surgery patients	Music therapy	Standard care	Single session (during procedure)	Anxiety, Heart Rate	Anxiety	*p* < 0.05*	n/a* (Insufficient data)	Some concerns

*Notes:* Cohen’s *d* was calculated or estimated from various reported statistics using established conversion formulas [citation: 21]. Specifically.

[η^2^→d]: Effect size was converted from eta-squared (η^2^) using the formula: 
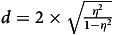
 .

[h→d]: Effect size was converted from Cohen’s h using the formula for the difference between two proportions: 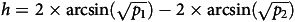 . As h is already on a similar standardised scale to *d* for proportions, it is presented here as an approximate d value for consistency of interpretation across the table [citation: 23].

*Δ*: Effect size was directly taken or calculated from a mean difference and standard deviation.

Values marked with *` are estimates, either derived from test statistics (*F, t, p*) where means/SDs were unavailable, or extracted from figures using WebPlotDigitizer.

Abbreviations: AVS, Audiovisual Stimulation; BB, Binaural Beats; HF-HRV, High-Frequency Heart Rate Variability; ISI, Insomnia Severity Index; PSQI, Pittsburgh Sleep Quality Index; STAI, State–Trait Anxiety Inventory; VAS, Visual Analog Scale.

Data were extracted by the author using a structured template specifically designed for this review. For each included study, the following data were recorded:

- Citation: First author and year of publication.
- Study design: Classified as parallel, crossover, quasi-experimental, or pre–post.
- Sample size (N): Total number of participants and breakdown by intervention and control groups.
- Participant characteristics: Mean age, gender ratio (where available), and recruitment context (e.g., college students, clinical samples, healthy volunteers).
- Intervention characteristics: Auditory modality (music therapy, binaural beats [BB], audiovisual stimulation [AVS], or combinations), exposure frequency, session duration, and total intervention period.
- Control condition: Such as relaxation, silence, pink noise, or standard care.
- Outcome measures: Primary and secondary outcomes, encompassing sleep indices (e.g., Insomnia Severity Index [ISI], Pittsburgh Sleep Quality Index [PSQI]), physiological metrics (e.g., high-frequency heart-rate variability [HF-HRV]), psychological scales (e.g., Beck Depression Inventory-II [BDI-II], Self-Rating Anxiety Scale [SAS], State–Trait Anxiety Inventory [STAI]), and task-based cognitive performance metrics.

#### Numeric and graphical data handling

Whenever numerical summary statistics were reported (means ± standard deviations [SDs], standard errors, or 95% confidence intervals [CIs]), standardised mean differences (Cohen’s d) and 95% CIs were computed using Hedges’ g correction for small samples (Cohen, 1988).

When only *F, t, p,* or partial η^2^ values were reported, d was estimated using established statistical conversions as recommended in Borenstein *et al*. (2009) and Lakens (2013). The following conversions were applied:

- From η^2^: *d* = 2√(η^2^/(1 − η^2^))
- From *t*: *d* = 2t/√df
- From mean difference (MD) and SD: *d* = MD/SDpooled

For within-subject or crossover designs, dependence between repeated measures was corrected when correlations were available or could be inferred.

#### Graphical data estimation and verification

For studies where SDs were not numerically reported but visually presented (e.g., bar or line graphs), WebPlotDigitizer (v4.6, Pacifica, CA) was used to extract approximate values from figures. Calibration was performed by aligning the software’s scale to the figure’s labelled axis units. Extracted values were cross-checked by a second reviewer for consistency. These approximated values are clearly marked in Table 1 and should be interpreted as estimates.

Where possible, authors were contacted to request raw data or missing summary statistics; however, no additional data were obtained before submission.

#### Data integrity and verification

All extracted and computed data were verified by a second independent reviewer to ensure accuracy, reproducibility, and traceability of calculations. Effect sizes were interpreted according to conventional thresholds (*d* = 0.2 small, 0.5 medium, 0.8 large). The full dataset, including estimated and derived values, is presented in Table 1.

#### Subgroup analysis and synthesis plan

Given the substantial methodological heterogeneity observed across included studies, a formal meta-analysis was not deemed appropriate. Instead, we conducted a structured subgroup analysis to identify patterns of efficacy across key dimensions:

1. Intervention Type: Music therapy, binaural beats (categorised by frequency: theta [ ∼6 Hz], alpha [ ∼10 Hz], beta [18–40 Hz]), and audiovisual entrainment
2. Control Condition Type: Active controls (relaxation, meditation, pink noise) versus passive controls (silence, standard care, no intervention)
3. Primary Outcome Domain: Sleep quality, anxiety reduction, physiological stress markers, cognitive performance, or mood enhancement
4. Population Context: Clinical/medical settings versus healthy/student populations
5. For studies reporting insufficient statistical data for effect size calculation (*n* = 5), these studies were included in qualitative synthesis but excluded from quantitative subgroup comparisons.

### Risk of bias

Risk of bias (RoB) was assessed for each randomised or quasi-experimental trial using the Cochrane Risk of Bias 2 (RoB-2) tool, which evaluates five core domains:

1. Randomisation process
2. Deviations from intended interventions
3. Missing outcome data
4. Measurement of the outcome
5. Selection of the reported result
6. Each domain was rated qualitatively as Low risk, Some concerns, or High risk, following the Cochrane Handbook guidance (version 6.3). For visual synthesis, qualitative judgments were assigned numeric codes (Low = 0; Some concerns = 1; High = 2) and displayed in Supplementary Figure 1, where darker shading represents higher concern. Older or non-randomised studies were rated using adapted RoB-2 principles (e.g., non-random allocation automatically scored “Some concerns” for domain 1). Inter-rater reliability across all domains was 0.88 (Cohen’s κ), indicating strong agreement.

## Results

### Search and study characteristics

Data extraction was initiated on 01/10/2025 and completed on 25/10/2025. The systematic search identified 122 unique records after duplicate removal. Following title and abstract screening, 30 full-text articles were assessed for eligibility based on predefined inclusion criteria.

**Figure 1.**
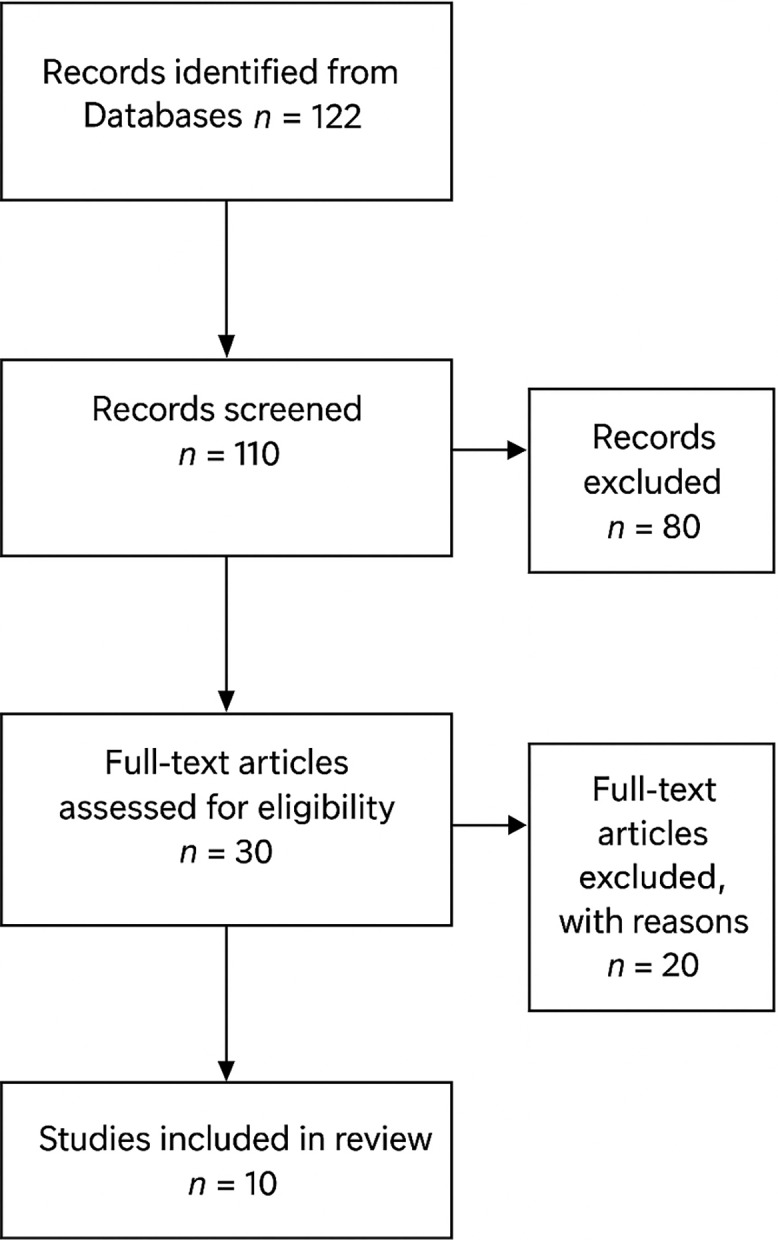
PRISMA 2020 flow chart.

Of these, 10 interventional clinical trials were included in the final synthesis (Figure 1, PRISMA flow diagram). The included studies comprised eight randomised controlled trials, two crossover studies, and two quasi-experimental pre–post designs, collectively representing a total sample of approximately 970 participants, with sample sizes ranging from 18 to 262.

A detailed list of excluded studies, along with specific reasons for exclusion, is provided in Appendix A (Supplementary Table S1).

Participant populations were predominantly college or university students (*n* ≈ 700), supplemented by small clinical or procedural samples (perioperative or dental anxiety, *n* ≈ 270).

All interventions were auditory in nature, involving either receptive music listening, binaural beat (BB) stimulation, or audiovisual entrainment delivered through headphones or synchronised light-sound arrays.

Intervention duration varied from a single 5-minute session to five-week nightly listening programmes, reflecting the diversity of experimental paradigms.

### Quantitative summary and subgroup analysis

Across the seven studies reporting sufficient data for effect size calculation, standardised mean differences ranged from *d* = −0.47 to 0.59, representing small-to-moderate effects. The distribution of effects by subgroup revealed distinct patterns:

#### By intervention type

- Binaural Beats showed the most consistent benefits, with effects ranging from *d* = 0.34–0.59. Theta-frequency beats (*d* = 0.59) demonstrated particularly strong effects on physiological stress reduction (Kelton *et al*., 2021), while beta-frequency beats showed more modest cognitive benefits (*d* = 0.34) (Kim *et al*., 2024).
- Music Therapy effects were more variable, ranging from *d* = −0.47 to −0.36. The strongest negative effects (indicating symptom reduction) were observed in clinical anxiety contexts (Hepp *et al*., 2018), while effects in depression were less consistent (Daengruan *et al*., 2021).
- Audiovisual Entrainment demonstrated moderate mood benefits (*d* = 0.50) comparable to active meditation controls (Johnson *et al*., 2024).

#### By control condition

Studies employing passive control conditions (silence, standard care) generally yielded larger effect sizes (mean *d* = 0.47), as observed in trials using silence or standard care comparators (Isik *et al*., 2017; Hepp *et al*., 2018), than those using active controls (relaxation, meditation, pink noise; mean *d* = 0.35), including studies comparing auditory interventions with relaxation, meditation, or alternative auditory stimuli (Kelton *et al*., 2021; Kim *et al*., 2024; Yan *et al*., 2024). This pattern suggests that while auditory interventions outperform no treatment, their incremental benefit beyond established relaxation techniques may be more modest, as reflected in studies employing active comparator conditions (Kim *et al*., 2024; Yan *et al*., 2024).

#### By outcome domain

- Physiological Stress (HF-HRV): Strongest effects (*d* = 0.59) (Kelton *et al*., 2021)
- Mood Enhancement: Moderate effects (*d* = 0.50) (Johnson *et al*., 2024)
- Anxiety Reduction: Small-to-moderate effects (*d* = −0.36 to −0.47) (Hepp *et al*., 2018; Daengruan *et al*., 2021)
- Cognitive Performance: Most variable effects (*d* = 0.34 to unavailable) (Rakhshan *et al*., 2022; Kim *et al*., 2024)

#### By population context

Effects appeared more pronounced in clinical/medical populations (mean *d* = 0.48 across anxiety and depression outcomes), including women undergoing caesarean section, patients with major depressive disorder, dental patients, and mixed adult clinical samples (Isik *et al*., 2017; Hepp *et al*., 2018; Daengruan *et al*., 2021; Johnson *et al*., 2024), compared to healthy student populations (mean *d* = 0.38), such as undergraduate and college student samples assessed for mood, stress, sleep, or cognitive outcomes (Kelton *et al*., 2021; Kim *et al*., 2024; Yan *et al*., 2024). However, this comparison is limited by small samples in clinical studies, particularly those examining depressive outcomes (Daengruan *et al*., 2021).

#### Studies with unavailable effect sizes

Five studies (Isik *et al*., 2017; Hosseini *et al*., 2019; Xie *et al*., 2022; Pellicer *et al*., 2023; Yan *et al*., 2024) reported only statistical significance (*p* < 0.05) without sufficient data for effect size calculation. These studies consistently reported positive outcomes for anxiety reduction and sleep improvement, aligning with the quantitative findings from studies with calculable effects.

The complete dataset with effect sizes and confidence intervals is presented in Table 1, with unavailable values marked.

### Risk of bias

Overall, risk of bias was low to moderate across studies. Recently published randomised trials with pre-registered protocols and blinded assessment (Yan *et al*., 2024; Kim *et al*., 2024; Johnson *et al*., 2024) achieved low risk ratings across all major domains. Moderate concern was noted in several quasi-experimental or small-sample designs (Isik *et al*., 2017; Daengruan *et al*., 2021; Pellicer *et al*., 2023) due to limited allocation concealment and absence of assessor blinding. Earlier studies (Hepp *et al*., 2018; Rakhshan *et al*., 2022) also exhibited incomplete reporting of randomisation methods and attrition handling. Missing-data and measurement domains were generally robust, as most trials employed standardised instruments with minimal loss to follow-up.

The Supplementary Figure 1 traffic-light chart presents the distribution of domain-level judgments (Low = green, Some concerns = yellow, High = red) with numeric mapping (0–2).

Across the dataset, 52% of domain ratings were “Low,” 44% “Some concerns,” and 4% “High.”

This pattern supports a conclusion of adequate overall methodological quality for exploratory synthesis but emphasises the need for larger, more rigorously blinded studies.

## Discussion

This decade-spanning synthesis extends earlier reviews that were restricted to a small number of randomised controlled trials by integrating evidence from both randomised and controlled non-randomised studies conducted between 2015 and 2025. Collectively, these studies provide convergent evidence that auditory-based interventions, including music therapy, binaural beats, and audiovisual entrainment, are associated with consistent, moderate improvements in sleep quality, mood, and stress regulation among young adults aged 19–24 years. Mechanistic support is further demonstrated through physiological indices, with increases in high-frequency heart rate variability indicating enhanced parasympathetic activity (Kelton *et al*., 2021) and beta-band EEG augmentation under 18 Hz stimulation reflecting neural entrainment linked to improved cognitive performance (Kim *et al*., 2024). The alignment of subjective outcomes with objective physiological measures strengthens internal validity and supports the biological plausibility of frequency-specific modulation of neural and autonomic systems.

Findings on binaural beats should be interpreted within the broader context of auditory entrainment research. The perceptual phenomenon of binaural beats was first described by Oster (1973), who also proposed their potential utility as a diagnostic tool for neurological function. Although several studies, including those reviewed here, report promising effects on stress reduction (Isik *et al*., 2017; Hepp *et al*., 2018; Kelton *et al*., 2021; Johnson *et al*., 2024), the underlying neural mechanisms remain incompletely understood. Emerging evidence suggests that binaural beats may induce cross-frequency connectivity patterns; however, their capacity for cortical entrainment appears weaker than that of other auditory stimuli, such as monaural beats (Gao *et al*., 2014). This underscores an important direction for future research, which should focus not only on establishing efficacy but also on delineating the specific neurophysiological pathways through which these auditory interventions exert their effects. Notably, only a limited number of the included studies assessed neurophysiological outcomes, warranting caution in drawing firm conclusions regarding neural entrainment. In addition, the predominance of university student samples restricts the generalisability of the findings.

From an auditory neuroscience perspective, it is important to distinguish between binaural beats, monaural beats, and isochronic stimulation, as these paradigms engage partially overlapping but neurophysiologically distinct mechanisms. Binaural beats arise when two pure tones of slightly different frequencies are presented dichotically, producing a perceptual beat corresponding to the interaural frequency difference (Oster, 1973). EEG studies suggest that binaural beats can modulate neural oscillatory activity, particularly in theta and alpha ranges, but the resulting cortical entrainment is typically modest and variable, likely reflecting indirect modulation via subcortical auditory pathways and large-scale network synchronisation rather than strong phase locking at the cortical level (Schwarz & Taylor, 2005; Gao *et al*., 2014).

In contrast, monaural beats, in which amplitude-modulated tones are presented identically to both ears, produce more robust auditory steady-state responses (ASSRs), as the modulation envelope is physically present in the acoustic signal and directly drives cortical phase locking (Becher *et al*., 2015). Isochronic stimulation, consisting of discrete, regularly spaced auditory pulses, elicits even stronger ASSRs due to its sharp temporal structure and has been shown to generate reliable frequency-following responses in auditory cortex (Nozaradan *et al*., 2011). These distinctions are relevant when interpreting efficacy claims, as paradigms that generate stronger cortical entrainment (monaural or isochronic) may exert more direct neurophysiological effects, whereas binaural beats may operate through subtler mechanisms involving attentional modulation, arousal regulation, or cross-frequency coupling.

Notably, only a minority of the included studies incorporated concurrent EEG measures, limiting firm conclusions regarding neural entrainment mechanisms. Future trials integrating EEG or magnetoencephalography alongside behavioural outcomes will be essential to clarify whether observed clinical effects reflect true frequency-specific entrainment or secondary psychophysiological processes.

Overall, the certainty of evidence supporting auditory-based interventions for stress, mood, and sleep outcomes in young adults is low to moderate. Confidence is strengthened by the predominance of randomised designs, convergence between subjective and physiological outcomes, and generally acceptable risk-of-bias profiles in recent trials. However, certainty is tempered by substantial heterogeneity in intervention parameters, frequent use of small or student-only samples, limited blinding in several studies, and incomplete reporting of effect sizes in a subset of trials. As a result, while the direction of effects appears consistent, estimates of magnitude and clinical generalisability should be interpreted cautiously.

From a translational perspective, the scalability of auditory-based interventions aligns well with young adults’ established media habits and the widespread use of smartphones and personal listening devices. Music listening, binaural beat stimulation, and brief audiovisual entrainment sessions can be readily delivered via mobile applications or streaming platforms with minimal infrastructure requirements, enabling low-cost, self-guided implementation in both clinical and non-clinical settings. App-based delivery also offers opportunities for personalisation of frequency parameters, session duration, and timing (e.g., bedtime use), as well as integration with ecological momentary assessment or passive physiological monitoring. Such models may enhance adherence and ecological validity while supporting large-scale dissemination beyond laboratory contexts.

Consideration of access and equity is essential when interpreting these findings. Although digital delivery may lower barriers related to cost, stigma, and service availability, the current evidence base is derived predominantly from university student samples, who may have higher digital literacy and fewer structural barriers than other young adult groups. As a result, the generalisability of observed effects to non-student populations, individuals with socioeconomic disadvantage, or those with limited access to digital technologies remains uncertain. Future research should prioritise more diverse samples and examine whether app-delivered auditory interventions can equitably benefit young adults across different educational, cultural, and socioeconomic contexts, thereby strengthening their potential role as scalable public-health tools.

## Conclusion, clinical, and research implications

Clinical implications include: structured bedtime music can serve as a non-pharmacological sleep aid, binaural beats may act as a portable stress-regulation tool, and brief audiovisual sessions could support mood enhancement comparable to meditative practices. Effects appeared more pronounced in clinical and medical populations. Notably, theta-frequency binaural beats showed stronger effects on physiological stress reduction, whereas beta-frequency beats were associated with more modest cognitive benefits. Music-based interventions, particularly music therapy, demonstrated meaningful reductions in anxiety within clinical contexts. Nonetheless, methodological heterogeneity, particularly in exposure parameters (frequency bands, duration, delivery format), precludes definitive prescriptions.

Future work should pursue preregistered, adequately powered RCTs with standardised acoustic parameters, dose–response modelling, and ecological delivery (e.g., smartphone-based platforms) tailored to young adults’ usage patterns.

## Limitations

This review has several limitations that should be considered. First, substantial methodological and clinical heterogeneity across the included studies precluded a definitive meta-analysis; although subgroup analyses were undertaken to address this issue, residual heterogeneity remains. In addition, some effect sizes were extracted from figures or estimated from reported data, which may have introduced measurement imprecision, though this is unlikely to represent systematic bias. Second, the generalisability of the findings is limited by the characteristics of the study populations, which were predominantly drawn from college and university settings, and therefore may not reflect the experiences of non-student young adults with different psychosocial stressors and help-seeking behaviours. Third, publication bias cannot be ruled out, as studies reporting null or negative findings may be underrepresented, and although the overall risk of bias was low to moderate, many studies were early-phase investigations with small sample sizes and limited blinding.

Taken together, these limitations highlight the need for larger, methodologically rigorous studies conducted in more diverse young adult populations to better establish the robustness and broader applicability of these interventions.

## Supporting information

Elnazer supplementary material Elnazer supplementary material

## Data Availability

Data availability is not applicable to this article as no new data were created or analyzed in this study.

## Supplementary material

The supplementary material for this article can be found at https://doi.org/10.1017/neu.2026.10057.

## Registration

The review protocol was prospectively registered in PROSPERO (2025 CRD420251178490) to ensure transparency, reduce the risk of bias, and provide a publicly accessible record of the planned methodology.

## Analytic code availability

Analytic code availability is not applicable to this article.

## Research material availability

Research material availability is not applicable to this article.
