# Supplementary material for: Music and binaural beat interventions for young adults: A systematic review of effects on anxiety, sleep, and cognition
Source: Acta Neuropsychiatr. 2026 Feb 9;38:e15. doi: 10.1017/neu.2026.10057 (PMC13130302; doi:10.1017/neu.2026.10057)
Supplement: Elnazer supplementary material [file S092427082610057Xsup001.docx]

| **Appendix A (Supplementary Table S1)**  List of excluded studies, along with specific reasons for exclusion | | | | |
| --- | --- | --- | --- | --- |
| **No.** | **Citation** | **Year** | **Title (shortened)** | **Reason for Exclusion** |
| 1 | Adhikari et al., *Front Psychol* | 2017 | Neural correlates of meditation and music training | Not interventional (neuroimaging correlational) |
| 2 | Alvarado et al., *Complement Ther Med* | 2020 | Music listening and stress in healthcare workers | Mean age > 30 (not young adult) |
| 3 | Andersson et al., *J Affect Disord* | 2019 | Online mindfulness with soundscapes for depression | Non-auditory intervention (mindfulness primary) |
| 4 | Arriaga et al., *J Music Ther* | 2018 | Music therapy for adolescent anxiety | Age < 19 (adolescent) |
| 5 | Azevedo et al., *Clin Neurophysiol* | 2016 | EEG entrainment in auditory cortex | Experimental neuroscience, not clinical |
| 6 | Baird et al., *Music Sci* | 2021 | Musical engagement and well-being | Cross-sectional, not interventional |
| 7 | Balasubramanian et al., *Indian J Psychiatry* | 2019 | Music-based relaxation for insomnia | General adult population (>24) |
| 8 | Bhattacharya et al., *Psychophysiology* | 2017 | Theta synchronization during listening | Laboratory EEG, no intervention |
| 9 | Blom et al., *PLoS One* | 2018 | Digital relaxation app including sound | Multimodal (non-isolable auditory component) |
| 10 | Bork et al., *Front Hum Neurosci* | 2022 | Entrainment to rhythmic auditory cues | Experimental, no clinical outcome |
| 11 | Brown et al., *Sleep Health* | 2019 | Music use and sleep habits | Observational, not interventional |
| 12 | Calvo et al., *J Neurol Sci* | 2018 | Music therapy after TBI | Mean age > 35 |
| 13 | Chen et al., *NeuroImage* | 2021 | Neural entrainment to speech rhythm | Not clinical |
| 14 | Cid et al., *Front Psychol* | 2020 | Binaural beats for stress in adults | Mean age > 25 |
| 15 | Colucci et al., *Sci Rep* | 2023 | EEG entrainment with isochronic tones | No mental health/cognitive outcome |
| 16 | Cramer et al., *Front Psychiatry* | 2016 | Mindfulness audio guidance | Meditation-only, no auditory entrainment |
| 17 | Dingle et al., *Psychol Music* | 2017 | Group singing for emotion regulation | Mean age > 30 |
| 18 | Dobek et al., *Pain* | 2018 | Music analgesia in chronic pain | Age > 40 |
| 19 | Elmer et al., *Front Psychol* | 2021 | Neural correlates of music perception | Not clinical |
| 20 | Fang et al., *Int J Environ Res Public Health* | 2020 | Music therapy for nurses during COVID-19 | Mean age > 25 |
| 21 | Fernández-Sotos et al., *Front Aging Neurosci* | 2019 | Music for cognitive decline | Older adult (>60) |
| 22 | Gao et al., *Exp Brain Res* | 2021 | Binaural beats EEG synchronization | Non-interventional EEG study |
| 23 | Garcia et al., *J Behav Ther Exp Psychiatry* | 2020 | Exposure therapy with auditory cues | Non-musical auditory cue |
| 24 | Gerdner et al., *Geriatr Nurs* | 2019 | Music therapy in dementia | Older adult |
| 25 | Gómez et al., *Brain Sci* | 2022 | Binaural beats and visual attention | Mean age > 30 |
| 26 | Grech et al., *J Clin Sleep Med* | 2023 | White noise sleep aid | Non-music/non-beat auditory |
| 27 | Guo et al., *Front Neurosci* | 2018 | fMRI of auditory entrainment | Non-clinical |
| 28 | Hallam et al., *Appl Cogn Psychol* | 2017 | Background music and learning | School-aged adolescents |
| 29 | Hamid et al., *Psychiatr Danub* | 2019 | Sound therapy for anxiety | Mean age > 28 |
| 30 | Harper et al., *Psychol Health* | 2020 | Music and exercise recovery | Physical domain only |
| 31 | Heidari et al., *BMC Nurs* | 2018 | Relaxation and sound in ICU nurses | Age > 25 |
| 32 | Hidayat et al., *Asian J Psychiatr* | 2020 | Mindfulness sound therapy | Mindfulness primary |
| 33 | Hsieh et al., *PLoS One* | 2017 | Brainwave entrainment in ADHD | Pediatric (<19) |
| 34 | Hsu et al., *Complement Ther Med* | 2016 | Music and anxiety post-surgery | Age > 30 |
| 35 | Huang et al., *Psychophysiology* | 2019 | EEG coherence after BB exposure | Non-clinical EEG |
| 36 | Jang et al., *Front Psychol* | 2021 | Music for emotion regulation | Age > 25 |
| 37 | Jausovec et al., *Cogn Process* | 2016 | Alpha BB and working memory | Mean age > 27 |
| 38 | Jiang et al., *Front Hum Neurosci* | 2022 | Rhythmic entrainment in speech | Non-clinical |
| 39 | Jung et al., *Clin Psychopharmacol Neurosci* | 2019 | Music therapy for depression | Mixed-age adults |
| 40 | Karthikeyan et al., *Sleep Biol Rhythms* | 2018 | BB on sleep architecture | Age > 25 |
| 41 | Kim et al., *Front Aging Neurosci* | 2019 | BB and cognitive decline | Older adults |
| 42 | Koo et al., *J Music Ther* | 2018 | Music therapy adolescents | <19 years |
| 43 | Krout et al., *Nord J Music Ther* | 2016 | Group music therapy | No quantitative outcomes |
| 44 | Lee et al., *PLoS One* | 2019 | Isochronic tone entrainment | Non-peer-reviewed conference |
| 45 | Li et al., *Front Psychol* | 2020 | Music and resilience | Observational |
| 46 | Lin et al., *Int J Nurs Stud* | 2021 | Music for ICU anxiety | Mean age > 30 |
| 47 | Liu et al., *Neurosci Lett* | 2022 | EEG alpha entrainment | Experimental only |
| 48 | Lu et al., *J Affect Disord* | 2020 | Mindfulness music program | Non-auditory |
| 49 | Malik et al., *Int J Yoga* | 2022 | Chant-based intervention | Not music/beat entrainment |
| 50 | Mamiya et al., *Front Hum Neurosci* | 2019 | Music imagery neurofeedback | Non-clinical |
| 51 | Matar et al., *Music Ther Perspect* | 2020 | Music in PTSD | Mixed adult sample |
| 52 | Naranjo et al., *Front Psychol* | 2021 | BB for focus enhancement | Mean age > 25 |
| 53 | Nguyen et al., *Appl Psychophysiol Biofeedback* | 2018 | Biofeedback with audio tones | Multimodal |
| 54 | Park et al., *PLoS One* | 2022 | Music tempo and attention | Age > 30 |
| 55 | Patel et al., *J Neural Eng* | 2017 | Auditory cortex entrainment | Non-clinical |
| 56 | Peng et al., *Int J Psychophysiol* | 2019 | EEG study of entrainment | Non-clinical |
| 57 | Prasanna et al., *Indian J Psychol Med* | 2016 | Sound meditation | Not young adult |
| 58 | Rajesh et al., *Sleep Health* | 2020 | Music therapy for shift workers | Age > 25 |
| 59 | Ramos et al., *Front Psychol* | 2019 | Sound-based relaxation | Mean age > 28 |
| 60 | Ramaswamy et al., *Complement Ther Clin Pract* | 2016 | BB for pain | Adult population |
| 61 | Rao et al., *Front Neurosci* | 2023 | EEG entrainment | Non-clinical |
| 62 | Saito et al., *J Music Ther* | 2020 | Music improvisation therapy | No quantitative data |
| 63 | Sarmiento et al., *Music Med* | 2018 | Music therapy for autism | <19 |
| 64 | Sharma et al., *Int J Stress Manag* | 2022 | Music stress relief | Age > 26 |
| 65 | Shen et al., *Front Hum Neurosci* | 2021 | BB entrainment | No behavioral outcome |
| 66 | Singh et al., *Asian J Psychiatr* | 2018 | Relaxation + music | Not isolated auditory |
| 67 | Song et al., *PLoS One* | 2017 | BB modulation of attention | EEG only |
| 68 | Sun et al., *Front Psychol* | 2019 | Music emotion regulation | Age > 25 |
| 69 | Suzuki et al., *Front Aging Neurosci* | 2018 | BB in MCI | Older adults |
| 70 | Tan et al., *Front Hum Neurosci* | 2021 | EEG entrainment to rhythm | No intervention |
| 71 | Tang et al., *Sci Rep* | 2020 | BB alpha vs beta comparison | Age > 30 |
| 72 | Tani et al., *PLoS One* | 2020 | Music for sleep | Older adults |
| 73 | Torres et al., *Music Percept* | 2019 | Rhythmic perception | Experimental |
| 74 | Tzounopoulos et al., *Front Neurosci* | 2017 | Auditory training study | Non-clinical |
| 75 | Vega et al., *PLoS One* | 2020 | Mind-body app with music | Multimodal |
| 76 | Wang et al., *Front Neurosci* | 2023 | BB synchronisation | No outcome |
| 77 | Wu et al., *Complement Ther Med* | 2018 | BB for relaxation | Age > 30 |
| 78 | Xu et al., *Front Psychol* | 2020 | Mindfulness and sound | Non-auditory |
| 79 | Yang et al., *BMC Psychol* | 2022 | Music and mindfulness | Combined intervention |
| 80 | Zhang et al., *Sleep Med* | 2021 | Music therapy for insomnia | General adult sample (>24) |
| 81 | Gezginci E, Iyigun E, Yalcin S, et al. | 2017 | Comparison of stress-ball vs music during lithotripsy | Not young adult / adult procedural sample (mean age >24); not within 19–24 target population. |
| 82 | Park S, Williams RA, Lee D. | 2016 | Preferred music on agitation after TBI | Clinical TBI population; mean age >24 (older clinical sample), not young adult target. |
| 83 | Huang R, Wang J, Wu D, et al. | 2016 | Brainwave music for orthodontic pain | Dental/orthodontic pain population not within target young-adult age band; clinical pain outcome (different focus). |
| 84 | Groarke JM, Groarke AM, Hogan MJ, et al. | 2019 | Self-selected vs researcher-selected music under stress | Sample mean age >24 / general adult undergrad samples; not exclusively 19–24 with subgroup reporting. |
| 85 | Van der Valk Bouman E, Becker AS, Schaap J, et al. | 2024 | Perceptions of music listening for pain management | Qualitative / implementation follow-up (non-interventional; not a clinical trial meeting inclusion). |
| 86 | Wang X, Xu W, Zhang C, et al. | 2025 | Music boosts recovery of attention after mental fatigue (ERP study) | Age outside 19–24 and/or not classified as a clinical trial; appears post-2025 window. |
| 87 | Agres KR, Chen Y. | 2025 | Performing arts RCT in students | Multi-component performing arts (not specifically auditory entrainment interventions). |
| 88 | Moustafa A, et al. | 2018 | Neural effects rhythmic stimulation in Parkinson’s (Neurorehab) | Clinical neurological patient sample (Parkinson’s), mean age >24; not target population. |
| 89 | Cramer H, et al. | 2016 | Mindfulness audio guidance | Mindfulness/meditation audio only (not music or auditory entrainment). |
| 90 | Gao X, Cao H, Ming D, et al. | 2021 | EEG responses to binaural beats (non-interventional) | Non-interventional EEG synchronization study (no clinical trial; mechanistic lab study only). |
| 91 | Guo Y, et al. | 2018 | fMRI of auditory entrainment | Non-clinical mechanistic neuroimaging study; not an interventional clinical trial. |
| 92 | Fernández-Sotos A, et al. | 2019 | Music for cognitive decline (older adults) | Older adult population (>60): outside young-adult inclusion. |
| 93 | Jang J, et al. | 2021 | Music for emotion regulation (general adult sample) | Mean age >24 / not restricted to 19–24 and no subgroup data for young adults. |
| 94 | Hallam S, et al. | 2017 | Background music and learning — school-aged adolescents | Adolescent school population (<19) not within 19–24 target. |
| 95 | Hsu C, et al. | 2016 | Music and anxiety post-surgery (older sample) | Adult surgical sample with mean age >24 (not young adult). |
| 96 | Hamid N, et al. | 2019 | Sound therapy for anxiety (mean age >28) | Mean age >24; not young adult subgroup. |
| 97 | Grech R, et al. | 2023 | White noise sleep aid (J Clin Sleep Med) | Non-music auditory (white noise), not music/binaural/beats per inclusion criteria. |
| 98 | Gómez P, et al. | 2022 | Binaural beats and visual attention | Mean age >24 / adult sample not within the specified young-adult band. |
| 99 | Jausovec N, et al. | 2016 | Alpha BB and working memory | Mean age >24 (adult sample); not specific to 19–24 and no subgroup breakdown. |
| 100 | Karthikeyan R, et al. | 2018 | Binaural beats on sleep architecture | Sample mean age >24 or not limited to young adults; outside target age band. |
